# An assessment of plant growth and N_2_ fixation in soybean genotypes grown in uninoculated soils collected from different locations in Ethiopia

**DOI:** 10.1007/s13199-018-0540-9

**Published:** 2018-02-22

**Authors:** Semira M. Beyan, Endalkachew Wolde-meskel, Felix D. Dakora

**Affiliations:** 1 School of Plant and Horticulture Science, Hawassa University, 05 Hawassa, Hawassa, Ethiopia; 2 International Livestock Research Institute Box, 5689 Addis Ababa, Ethiopia; 3 Present Address: Department of Chemistry, Tshwane University of Technology, Private Bag X680, Pretoria, 0001 South Africa

**Keywords:** Rhizobia, %Ndfa, Soil

## Abstract

Achieving food and nutritional security is a major challenge in Ethiopia, especially with increasing human population and low crop productivity. Legumes offer an alternative choice to chemical fertilizers for increasing crop yields. The aim of this study was to assess, under glasshouse conditions, plant growth and symbiotic performance of uninoculated soybean genotypes planted in soils collected from different locations in Ethiopia. The results showed significant differences in plant growth and symbiotic performance among the soybean genotypes planted in different soils. There was a location-specific effect of soil on plant growth and symbiotic N nutrition of soybean. Whole-plant biomass was highest in soil from Amaro, followed by Boricha, Dorebafano, Pawe, and Mambuk. The δ^15^N values ranged from +0.82‰ for Pawe to +5.11‰ at Dorebafano. However, %Ndfa of soybean was greater in plants grown in Mambuk soil, followed by Pawe with the lowest %Ndfa being in Amaro soil. The amount of N-fixed followed similar pattern as %Ndfa. The significant interaction found between soil type and soybean genotype for plant DM, shoot N concentration, δ^15^N, %Ndfa, N-fixed and soil N-uptake clearly indicated the effect of soil factors. This study revealed the presence of native rhizobia in Ethiopian soils that are compatible with soybean. The N contribution of the soybean genotypes was variable, and strongly influenced by the soil factors.

## Introduction

Industrially, legumes are used to prepare biodegradable plastics, oils, gums, dyes and ink (Graham and Vance 2003*).* Legumes also have the ability to fix atmospheric N_2_ when in symbiosis with soil rhizobia. The annual fixed-N has been estimated to be 21.45 Tg from food legumes, and 12–25 Tg from pasture fodder legumes (Herridge et al. 2008). In Ethiopia, legume crops such as lentil, faba bean, common bean and chick pea constitute the staple food of the people and are part of the local culinary culture. Additionally, legumes are a valuable source of N-rich feed for livestock and high protein diet for humans. Furthermore, legumes are cultivated as an intercrop, or in rotation with cereal crops (e.g. maize, sorghum and millet) in order to restore soil fertility. Despite their importance, legume crop yields have remained low relative to cereals, especially in developing countries (Graham and Vance 2003; Siddique et al. 2011) due largely to low funding for legume research in Africa.

Soybean is the most important food grain legumes in the world, as the grain contains 40% protein, 26% carbohydrate and 20% oil in addition to being rich in vitamins, fibre and minerals (Wasike et al. 2009). It is estimated that N_2_ fixation by soybean alone represents 77% of the N-fixed by crop legumes (Herridge et al. 2008). About 50–60% or more of the N requirement of soybean is met from BNF (Salvagiotti et al. 2008; Mapope and Dakora, 2016). The high level of N-fixed could be of major benefit to cropping systems in African, where soils are inherently low in nutrients and there is little use of chemical fertilizers (Dakora and Keya 1997; Sinclair et al. 2014). Despite these advantages, soybean production has remained very low in Africa (Maingi et al. 2006; Abate et al. 2012).

Low soil N concentration is a major factor limiting crop production, in Sub-Saharan African countries that includes Ethiopia. In modern agriculture, nitrogen fertilization is widely used to improve crop yields. However, most farmers in Ethiopia cannot afford or access mineral fertilisers to improve crop N nutrition. As a result they practice low N-input agriculture. Hence, the N contribution by the legume-rhizobia symbiosis could be very significant to Ethiopian farmers.

Although soybean is a new crop in Ethiopia, its production is rapidly increasing to meet industry demands and overcome protein-calorie malnutrition. That notwithstanding, Ethiopia is still one of the largest soybean importing countries in Sub-Saharan Africa (Mutegi and Zingore 2014). There is therefore a need to increase soybean production locally and this can be done through identifying soybean genotypes that are high-yielding, high N_2_-fixing and can contribute substantially to soil N fertility through effective symbiosis with native rhizobia.

Studies done so far on soybean in Ethiopia have focused on rhizobial inoculation response, genetic variability of soybean for agronomic traits, and intercropping trials with cereals (Bekere et al. 2013; Dilnesaw and Getahun 2013; Zerihun et al. 2014; Jaiswal et al. 2016). Little information currently exists on i) the presence or absence of effective native soybean rhizobia in Ethiopian soils, ii) the response of different soybean genotypes to nodulation and N_2_ fixation by indigenous rhizobia in Ethiopia, and iii) the distribution of rhizobia in Ethiopian soils. The aim of this study was i) to assess the ability of native rhizobia to nodulate soybean, as well as ii) to assess plant growth and symbiotic performance of soybean genotypes planted in uninoculated soils collected from different locations in Ethiopia under glasshouse conditions.

## Materials and methods

### Site description

The soil samples used in this study were collected from five locations (namely, Amaro, Boricha, Dorebafano, Mambuk and Pawe) within the soybean-growing areas of Ethiopia. maro, Boricha and Dorebafano are located in the Southern Nations Nationalities and People’s Region (SNNPR) of Ethiopia, an area found between geographical coordinates 4^°^ 43′ and 8^°^ 58′ N and 34^°^ 88′ and 39^°^ 14′ E. The SNNPR region experiences annual rainfall ranging from 400 to 2200 mm and daily average temperature of 10 to 27 (http://www.snnprs.gov.et/about.html). However, Mambuk and Pawe are located in the Benshangul Gumuz Region (BGR) with coordinates 10° 38′ N and 35° 43′ E in north-eastern Ethiopia. The BGR is close to the Ethiopia-Sudan border, and is characterized by 500 to 1800 mm of annual rainfall, 21 to 35 °C daily temperature and 600 to 2731 m above sea level.

#### Soil collection

Fields with no history of rhizobial inoculation were particularly targeted for soil collection. Soils were sampled at 20-cm depth, placed in plastic bags and taken to the laboratory, where they were air-dried in a dust-free environment and sieved (5 mm). A sub-sample of the sieved soil was analysed at the Agricultural Research Council laboratory in Pretoria, South Africa, for pH, electrical conductivity (EC), organic carbon, total N, cation exchange capacity (CEC) and plant-available P. At the laboratory, the sieved soil from each location was used to fill 4-L capacity plastic pots that were previously surface-sterilised. All potted soils were supplied with a blanket treatment of 46 kg P ha^−1^ as triple superphosphate (TSP) before planting.

### Seed source, seed sterilisation and planting

Eleven soybean genotypes (namely, AGS-71, AFGAT, Awassa-95, Coker-240, Clark-63 K, Crowford, Gishama, Nova, TGx-3326-44, Wegayen and Williams) obtained from the Awassa and Pawe Agricultural Research Centres in Ethiopia, were used in this study. Seeds of non-legume species included as reference plants were obtained from Hawassa University, Ethiopia. Where there was history of soybean cultivation, the soybean genotypes used by farmers, were planted at those particular locations. The exception was forest soil which had no history of soybean cultivation.

Soybean seeds were surface-sterilized (Vincent 1970) and the pre-germinated seedlings planted in potted soil (Somasegaran & Hoben, 1985, Woomer et al., 2011). Three replicate pots were used for each soybean genotype. The 3 non-legume species (*Sorghum bicolour* (L.), *Eragrostis tef*, *Triticum aestivum)* included as reference plants were similarly processed and planted in triplicates to measure soil N uptake. The plants were raised under natural light in the glasshouse.

#### Plant harvest and processing

At 58 days after planting (DAP) at R1 stage, the soybean plants were harvested and separated into shoots, roots and nodules. The shoots and roots were oven-dried at 60 °C for 48 h and weighed. The shoots of reference plants were also harvested, oven-dried and weighed. The shoots of both legume and reference plants were each milled separately to a fine powder (0.85 mm size) and stored prior to ^15^N isotopic analysis.

### ^15^N/^14^N isotopic analysis

The finely ground (0.85 mm) shoot samples of soybean and reference plants were analysed for ^15^N/^14^N ratio and %N using mass spectrometry. Plant samples were weighed into Al capsules (2.0–2.5 mg/sample) and each fed into a Carlo Erba NA1500 elemental analyser (Fisons Instruments SpA, Strada, Rivoltana, Italy) coupled to a Finnigan MAT252 mass spectrometer via conflo II open-split device. The ^15^N abundance, usually expressed in a relative δ (delta) notation, is the ‰ deviation of the ^15^N natural abundance of the sample from atmospheric N_2_ (0.3667 atom% ^15^N). The isotopic composition (δ^15^N) of sample was measured as (Mariotti 1983):

$$ {\updelta}^{15}\mathrm{N}\left({\mbox{\fontencoding{U}\fontfamily{wasy}\selectfont\char104}} \right)=\frac{{\left[{}^{15}\mathrm{N}/{}^{14}\mathrm{N}\right]}_{\mathrm{sample}}-{\left[{}^{15}\mathrm{N}/{}^{14}\mathrm{N}\right]}_{\mathrm{standard}}}{{\left[{}^{15}\mathrm{N}/{}^{14}\mathrm{N}\right]}_{\mathrm{standard}}}\times 1000 $$

Where, ^15^N/^14^N_sample_ and ^15^N/^14^N_standard_ are the abundance ratios of the plant and air as standard, respectively.

The percent N derived from atmospheric N_2_ (%Ndfa) was quantified as (Shearer & Kohl, 1986; Unkovich et al. 2008):

$$ \%\mathrm{Ndfa}=\frac{\left[{\updelta}^{15}{\mathrm{N}}_{\mathrm{ref}}-{\updelta}^{15}{\mathrm{N}}_{\mathrm{leg}}\right]}{\left[{\updelta}^{15}{\mathrm{N}}_{\mathrm{ref}}-\mathrm{B}\ \mathrm{value}\right]}\mathrm{x}\ 100, $$

Where δ^15^N_ref_ is the ^15^N natural abundance of non-N_2_ fixing reference plant, δ^15^N_leg_ is the ^15^N natural abundance of the legume (soybean), and B value is ^15^N natural abundance of soybean plants which were solely dependent on symbiotic N_2_ fixation for their N nutrition. The mean δ^15^N values of the reference plants for each location were used to estimate the percent N derived from the atmosphere in soybean plants.

The amount of N-fixed was calculated as (Maskey et al., 2001):

$$ {\mathrm{N}}_{\mathrm{fixed}}=\%\mathrm{Ndfa}\ \mathrm{x}\ \mathrm{Legume}\ \mathrm{shoot}\ \mathrm{N}\ \mathrm{content} $$

The N content of shoots was determined as the product of %N and sample dry weight (Pausch et al., 1996).

### Statistical analysis

Statistical analysis was performed using STATISTICA (StaSoft Inc., Tulsa, OK, USA) package. Plant growth and isotopic data were tested for normality in distribution and then subjected to a one-way analysis of variance (ANOVA) for each location, and a 2-Way ANOVA to compare the means of results between the two regions. Where there was a significant difference, the means were separated using the Duncan’s multiple range test at *p* ≤ 0.05.

## Results

### Soil chemical properties

The soil samples collected from each of the study sites (Amaro, Boricha, Dorebafano, Mambuk and Pawe) were analysed for pH, organic carbon (OC), electrical conductivity (EC), cation exchange capacity (CEC), total N and plant-available P (Table 1). The pH of soils from the study sites ranged from pH 5.46 to 6.18. The EC of soils was low and ranged from 1 to 20 mS m^−1^. The organic carbon content ranged from 1.29 to 2.15%. The plant available P ranged from 0.35 to 24.7 mg kg^−1^. According to the Bray-1 P test rating, the soil from Dorebafano (24.7 mg kg^−1^) had medium P fertility, Boricha (10.2 mg kg^−1^) low P, Amaro (6.7 mg kg^−1^), Mambuk (0.35 and 0.44 mg kg^−1^) and Pawe (0.60 mg kg^−1^) very low P (Jones, 2002). According to Havlin et al. (1999), soil fertility can be classified according to the soils total nitrogen content (%), as very low (<0.1), low (0.1–0.15) medium (0.15–0.25), and high (>0.25). The soils from all the study sites contained low soil total nitrogen, except for Mambuk forest which had medium N concentration (0.188%). Nitrate levels of the soils were low, except for Mambuk and Amaro with moderate concentrations. The CEC of soils from Pawe (32.67) and Mambuk (37.90) were high, while the rest showed a moderate CEC (cmol (+) kg^−1^) (Table 1; see Hazelton & Murphy, 2007).

**Table 1:** Chemical properties of soils from the study sites

Locations	Soil properties						
	pH	EC	OC	P	Total N	N-NO_3_	CEC
		mS m^−1^	%	mg kg^−1^	%	mg kg^−1^	cmol(+) kg^−1^
Mambuk	6.10	5	1.77	0.44	0.122	15.67	37.9
Mambuk forest soil	5.83	2	1.77	0.35	0.188	4.98	23.84
Dorebafano	6.18	20	1.29	24.7	0.115	4.04	14.23
Amaro	6.08	2	2.15	6.7	0.123	12.19	21.59
Pawe	5.57	1	1.94	0.60	0.117	2.82	32.67
Boricha	5.46	2	1.69	10.2	0.104	1.52	15.44
Method of analysis	pH(H_2_O)	1:10 water extract	Walkley black	P-Bray1	Total N digest	KCl extract	Ammo. acetate

### δ^15^N of reference plant species and B value

Estimating the percentage N derived from the atmosphere (%Ndfa) by nodulated legumes using the ^15^N natural abundance technique requires a value for soil N uptake by the legume. In this study, *Sorghum bicolour* (L.), *Eragrostis tef* and *Triticum aestivum* were used as reference plants. The δ^15^N values varied from +2.21‰ for *Triticum aestivum* in Pawe soil to +10.46‰ for *Eragrostis tef* in Mambuk soil. The average δ^15^N for the 3 reference plants for each site ranged from +2.72‰ for Pawe to +6.33‰ for Dorebafano (Table 2). Since there was no measured local B value for soybean in Ethiopia, a previously published average B value of −1.83‰ was used (Unkovich et al. 2008).

**Table 2:** δ^15^N values (‰) of non-fixing reference plants used for estimating soil N-uptake by soybean genotypes in Ethiopia in 2012

Reference plant	δ^15^N (‰) values of reference plants in soils from study sites					
	Pawee	Mambuk	Amaro	Boricha	Dorebafano	Mambuk Forest soil
*Sorghum bicolour* (L.)	2.46	5.48	5.11	6.11	6.11	3.51
*Eragrostis tef*	3.47	10.46	3.93	5.47	5.47	3.82
*Triticum aestivum*	2.21	2.11	6.72	5.78	7.41	4.25
Average	2.72	6.01	5.25	5.98	6.33	3.86

### Plant growth, shoot N concentration and content, and symbiotic performance of soybean genotypes at each location

#### Benshangul Gumuz region

##### Pawe

At Pawe, a 1-Way ANOVA showed significant differences in plant growth and symbiotic performance between and among the six soybean genotypes.

Shoot, root and whole-plant dry matter at Pawe varied significantly among the genotypes (Table 3). Shoot DM ranged from 3.30 g plant ^−1^ for the genotype Coker-240 to 5.66 g plant^−1^ for TGx-3326-44 (Table 3). Although the root DM was similar for the test soybean genotypes, TGx3326–44, AGS-71 and Wegayen produced the most whole-plant biomass (Table 3).

**Table 3:** Symbiotic performance of soybean varieties grown in soils collected from Pawe, Mambuk, Mambuk forest, Amaro, Boricha and Dorebafano, in Ethiopia, 2012

Soil location	Genotypes	Drymatter (DM)			Shoot N concentration	Shoot N content	δ^15^N	Ndfa	N-fixed	Soil N-uptake
		Shoot	Root	Whole plant						
			g plant^−1^		%	mg plant^−1^	‰	%	mg plant^−1^	mg plant^−1^
BGR region										
Pawe	AGS-71	5.63 ± 0.88a	1.36 ± 0.19a	6.99 ± 0.74a	1.95 ± 0.13b	107.3 ± 8.8b	0.25 ± 0.24c	54.3 ± 5.3a	57.4 ± 2.1ab	50.0 ± 10.0b
	Coker-240	3.30 ± 0.08c	1.38 ± 0.46a	4.69 ± 0.53c	2.73 ± 0.23a	90.6 ± 9.4b	1.87 ± 0.39a	18.7 ± 4.0c	16.2 ± 1.7c	74.4 ± 11.0ab
	Gishama	3.95 ± 0.01bc	1.09 ± 0.18a	5.05 ± 0.18bc	2.29 ± 0.13ab	90.5 ± 4.9b	0.56 ± 0.06c	47.5 ± 1.2a	43.2 ± 3.3b	47.4 ± 1.6b
	Nova	5.25 ± 0.22a	1.08 ± 0.18a	6.33 ± 0.35ab	2.68 ± 0.09a	140.5 ± 7.1a	1.32 ± 0.32ab	30.8 ± 7.1bc	43.4 ± 9.9b	97.1 ± 10.0a
	TGx-3326-44	5.66 ± 0.13a	1.47 ± 0.11a	7.13 ± 0.24a	1.92 ± 0.08b	108.7 ± 3.0b	0.13 ± 0.35c	57.0 ± 7.7a	61.6 ± 7.5a	47.1 ± 9.5b
	Wegayen	4.86 ± 0.15ab	1.59 ± 0.13a	6.45 ± 0.20a	2.18 ± 0.18b	105.4 ± 6.3b	0.77 ± 0.17bc	42.9 ± 3.7ab	44.6 ± 1.1ab	60.8 ± 7.4b
	F- statistics	6.35**	0.71 ns	5.70**	5.57**	6.84*	7.62**	7.60**	8.69***	5.03**
Mambuk	Clark-63 K	2.80 ± 0.25b	1.06 ± 0.15ab	3.86 ± 0.38b	2.49 ± 0.08a	69.9 ± 6.8a	1.84 ± 0.02b	53.2 ± 0.3b	37.2 ± 3.8bc	32.7 ± 3.0bc
	Crowford	3.84 ± 0.12a	1.45 ± 0.08a	5.29 ± 0.20a	2.32 ± 0.08ab	88.9 ± 3.2a	1.97 ± 0.35b	51.4 ± 4.5b	45.5 ± 3.5abc	43.4 ± 5.0b
	Gihsama	3.87 ± 0.24a	0.79 ± 0.10bc	4.59 ± 0.16ab	2.15 ± 0.13ab	82.0 ± 9.7a	0.87 ± 0.10c	65.6 ± 1.2a	53.8 ± 5.5ab	28.6 ± 4.2c
	Nova	4.21 ± 0.19a	0.62 ± 0.15c	4.83 ± 0.32ab	2.10 ± 0.15ab	88.1 ± 5.5a	3.41 ± 0.53a	33.1 ± 6.8c	28.5 ± 3.9c	59.6 ± 9.3a
	TGx-3326-44	4.35 ± 0.37a	0.84 ± 0.12bc	5.19 ± 0.49a	1.91 ± 0.19b	82.6 ± 8.5a	0.52 ± 0.42c	69.9 ± 5.4a	58.4 ± 9.4a	24.1 ± 2.7c
	F-statistics	5.01*	6.5**	2.96*	2.39*	ns	18.20***	18.20***	4.10*	10.72**
Mambuk Forest soil	AGS-71	4.69 ± 0.21ab	0.94 ± 0.18a	5.6 ± 0.10bcd	1.99 ± 0.07a	93.6 ± 7.4 cd	0.08 ± 0.09c	66.5 ± 1.6a	62.1 ± 3.9ab	31.5 ± 3.8c
	Crowford	4.34 ± 0.17 cd	0.93 ± 0.10a	5.3 ± 0.17bcd	2.20 ± 0.03a	95.1 ± 2.8 cd	2.47 ± 0.08a	24.4 ± 1.3d	23.2 ± 1.1d	71.9 ± 2.9b
	Williams	3.74 ± 0.20d	1.09 ± 0.04a	4.8 ± 0.23d	2.46 ± 0.22a	91.6 ± 8.5d	1.24 ± 0.88abc	46.1 ± 15.4c	41.7 ± 2.5bcd	51.6 ± 6.7bc
	Coker-240	3.80 ± 0.17d	1.14 ± 0.22a	4.9 ± 0.36 cd	2.41 ± 0.19a	92.0 ± 10.0d	1.83 ± 0.63ab	35.7 ± 10.9 cd	35.0 ± 14 cd	57.0 ± 5.2b
	AFGAT	4.76 ± 0.39bc	1.25 ± 0.13a	6.0 ± 0.52bc	2.55 ± 0.11a	120.7 ± 4.7b	0.59 ± 0.17bc	57.5 ± 2.9ab	69.2 ± 1.9a	49.8 ± 5.4bc
	Awassa95	7.54 ± 0.23a	1.01 ± 0.17a	8.5 ± 0.393a	1.91 ± 0.56a	143.6 ± 6.4a	2.23 ± 0.54a	28.7 ± 9.4d	41.5 ± 14bcd	101.4 ± 13a
	Wegayen	5.16 ± 0.25b	1.09 ± 0.18a	6.3 ± 0.42b	2.23 ± 0.04a	115.4 ± 7.3bc	0.91 ± 0.62abc	51.8 ± 10.8b	59.3 ± 2.4abc	56.0 ± 6.1b
	F-statistics	27.4***	0.53 ns	13.7***	0.9 ns	7.87***	7.10**	7.09**	4.30**	10.18***
SNNPR										
Amaro	Awassa95	5.76 ± 0.38a	1.96 ± 0.31a	7.72 ± 1.04a	1.89 ± 0.08a	107 ± 9a	4.87 ± 0.09a	5.37 ± 1.28d	6.0 ± 0.8b	101.0 ± 8.0ab
	Clark-63 K	5.53 ± 0.25a	1.54 ± 0.15ab	7.07 ± 0.22a	1.96 ± 0.04a	108.6 ± 6.32a	4.04 ± .004 cd	17.04 ± 0.58ab	18.5 ± 1.2a	90.1 ± 5.2abc
	Crowford	5.22 ± 0.17a	1.11 ± 0.11b	6.33 ± 0.25a	1.75 ± 0.10a	91.1 ± 2.0a	4.25 ± 0.08bc	14.08 ± 1.10bc	12.8 ± 0.9ab	78.0 ± 2.0abc
	Gishama	5.36 ± 0.24a	1.87 ± 0.10ab	6.73 ± 0.32a	1.76 ± 0.12a	85.2 ± 2.0a	4.01 ± 0.08 cd	17.56 ± 1.11ab	14.9 ± 1.8a	70.0 ± 9.0bc
	Nova	6.28 ± 0.34a	1.47 ± 0.12ab	7.74 ± 0.23a	1.56 ± 0.13a	97.0 ± 5.9a	4.20 ± 0.13bc	14.78 ± 1.89bc	14.5 ± 2.4ab	82.6 ± 4.3abc
	TGx-3326-44	5.45 ± 0.42a	2.08 ± 0.12a	8.03 ± 0.82a	1.51 ± 0.12a	88.3 ± 10.0a	3.67 ± 0.11d	22.27 ± 1.59a	19.5 ± 2.3a	68.8 ± 2.0c
	Williams	6.45 ± 0.33a	1.33 ± 0.11ab	7.78 ± 0.23a	1.85 ± 0.27a	118.0 ± 13.0a	4.55 ± 0.07ab	9.93 ± 0.98 cd	12.0 ± 1.9ab	106.2 ± 10a
	F-statistics	2.37 ns	4.76**	1.37 ns	1.45 ns	2.38 ns	18.38***	18.37***	6.58***	4.89**
Boricha	AGS-71	4.41 ± 0.08a	1.46 ± 0.16b	5.87 ± 0.24a	2.33 ± 0.14a	102.9 ± 7.9a	4.30 ± 0.06b	21.51 ± 0.78a	22.17 ± 2.0a	80.7 ± 6.02b
	Awassa95	4.45 ± 0.62a	1.88 ± 0.43ab	6.33 ± 1.03a	2.27 ± 0.04a	100.9 ± 13.3a	5.01 ± 0.16a	12.38 ± 2.0b	13.02 ± 3.8b	87.9 ± 9.53ab
	Coker-240	4.75 ± 0.79a	2.43 ± 0.24a	7.18 ± 0.99a	2.66 ± 0.16a	125.5 ± 20.4a	4.44 ± 0.23b	19.72 ± 2.9a	25.46 ± 6.6a	100.1 ± 14.38a
	Wegayen	3.90 ± 0.14a	1.84 ± 0.14ab	5.74 ± 0.19a	2.39 ± 0.20a	92.9 ± 5.52a	3.99 ± 0.15b	25.52 ± 1.9a	23.54 ± 1.1ab	69.3 ± 5.71ab
	Williams	4.18 ± 0.16a	1.83 ± 0.12ab	6.01 ± 0.10a	2.65 ± 0.21a	110.5 ± 9.05a	5.05 ± 0.17b	11.95 ± 2.2b	12.81 ± 1.4b	97.7 ± 10.48ab
	F-statistics	0.48 ns	2.04 ns	0.77 ns	1.27 ns	0.99 ns	8.18**	8.18**	2.79*	1.68*
Dorebaf-ano	AFGAT	4.56 ± 0.48a	1.68 ± 0.13a	6.24 ± 0.13a	2.68 ± 0.18a	122.3 ± 15.8a	5.05 ± 0.03b	16.90 ± 0.49b	20.70 ± 2.86a	101.5 ± 13.0a
	Crowford	4.00 ± 0.19a	1.98 ± 0.40a	5.98 ± 0.40a	2.32 ± 0.13ab	92.2 ± 0.8ab	5.77 ± 0.14a	7.50 ± 1.89c	6.9 ± 1.7b	85.3 ± 2.5ab
	Gishama	3.87 ± 0.03a	2.06 ± 0.24a	5.93 ± 0.26a	2.04 ± 0.21b	79.1 ± 8.7b	5.30 ± 0.19b	13.71 ± 2.53b	11.3 ± 3.4b	67.8 ± 5.3b
	TGX-3326-44	4.26 ± 0.24a	2.15 ± 0.16a	6.41 ± 0.40a	2.05 ± 0.18b	86.6 ± 4.7b	4.32 ± 0.08c	26.80 ± 1.07a	23.1 ± 0.8a	63.5 ± 4.2b
	F-statistics	1.13 ns	0.63 ns	0.36 ns	2.87*	4.11*	22.89***	22.89***	10.19**	5.48*

Values (Mean ± SE) with dissimilar letters in a column are significantly different at *p* ≤ 0.05 (*), *p* ≤ 0.01 (**), *p* ≤ 0.001 (***)

Shoot N concentration also ranged from 1.92% for genotype TGx-3326-44 to 2.73% for Coker-240. Genotypes AGS-71 and TGx-3326-44 recorded the lowest shoot N concentration, 1.92 and 1.95% respectively (Table 3). Shoot N content was highest in genotype Nova (140.5 mg plant^−1^), followed by TGx-3326-44 (108.7 mg plant^−1^), AGS-71 (107.3 mg plant^−1^), Wegayen (105.4 mg plant^−1^), Coker-240 (90.6 mg plant^−1^) and least in genotype Gishama (90.5 mg plant^−1^).

Shoot δ^15^N values of soybean genotypes also differed when planted in soil from Pawe, with genotypes TGx-3326-44 (+0.13) and AGS-71 (+0.25‰) exhibiting the lowest δ^15^N values, followed by Gishama (+0.56‰) and Wegayen (+0.77‰). Genotype Coker-240 and Nova recorded the highest δ^15^N values, +1.87‰ and 1.32‰ respectively. As a result, the %Ndfa of TGx-3326-44 and AGS-71 (which showed lower δ^15^N) were much higher, 57.0 and 54.3%, respectively (Table, 3). By contrast, Coker-240 which had the highest δ^15^N (+1.87‰) recorded the lowest %Ndfa (18.7%). The soybean genotypes varied in the amounts of N-fixed, which ranged from 16.2 mg plant^−1^ for Coker-240 to 61.6 mg plant^−1^ for TGx3326–44. Soil N-uptake was highest for Nova (97.1 mg plant^−1^) and lowest (47.1 mg plant^−1^) for TGx3326–44 (Table 3).

##### Mambuk

The genotypes assessed for plant growth and N-nutrition at Mambuk included TGx-3326-44, Gishama, Clark-63 K, Nova and Crowford.

Of these five genotypes, TGx-3326-44 (4.35 g.plant^−1^) Nova (4.2 g.plant^−1^), Gishama (3.87 g.plant^−1^) and Crowford (38.4 g.plant^−1^) produced much greater shoot DM compared to Clark-63 K (2.80 g.plant^−1^). Root DM was highest for Crowford, followed by genotype Clark-63 K (Table 3). Genotype Crowford also had the highest whole-plant biomass, followed by genotype TGx-3326-44, Nova and Gishama. Shoot N concentration varied from 1.91% for genotype TGx-3326-44 to 2.49% for Clark-63 K. Shoot N content was similar for the five test genotypes (Table 3).

Shoot δ^15^N was lowest (+0.52‰) in genotype TGx-3326-44 and highest (+3.41%) in Nova. As a result, TGx-3326-44 recorded the highest %Ndfa (69.9%), followed by genotype Gishama (65.6%), with Nova (33.1%) being the lowest. The highest N contribution was by genotype TGx-3326-44 (58.4 g.plant^−1^), followed by Gishama and Crowford, 53.8 and 45.5 mg.plant^−1^, respectively (Table 3). Soil N-uptake by these genotypes ranged from 24.1 mg plant^−1^ for TGx-3326-44 to 59.6 mg plant^−1^ for Nova. The genotypes with higher amounts of N- fixed, took up less soil N (Table 3).

##### Mambuk forest

At Mambuk forest, 11 soybean genotypes were planted in soil collected from undisturbed fields with no history of soybean cultivation. Of the 11 genotypes, only seven (AGS-71, Awassa-95, Coker-240, Crowford, Gishama, Wegayen, and Williams) successfully nodulated with native soil rhizobia. (Table 3).

Awassa −95 produced the largest shoot biomass, followed by Wegayen, and lowest in Williams and Coker-240. Root DM was similar for all seven genotypes, but whole-plant biomass was significantly greater in Awassa-95, followed by Wegayen, and AFGAT, and lowest in Williams. Although shoot N concentration was also similar for all genotypes, N content was highest in Awassa-95, followed by AFGAT, and lowest in Williams (Table 3).

The shoot δ^15^N of soybean genotypes ranged from +0.08‰ for AGS-71 to +2.47‰ for Crowford. As a result, AGS-71 and Crowford had the highest (66.5%) and the lowest (24.4%) %Ndfa, respectively. The amount of N-fixed was much greater in genotype AFGAT (69.2 mg plant^−1^), followed by AGS-71 (62.1 mg plant^−1^) and Wegayen (59.3 mg plant^−1^), and lowest in Crowford (23.6 mg plant^−1^). Soil N-uptake was significantly increased in Awassa-95, followed by Crowford, and lowest in AGS-71 (Table 3).

#### Southern nations nationalities and People’s region (SPNNR)

##### Amaro

At Amaro shoot biomass was similar for the seven soybean genotypes tested, and because the differences in root dry matter were small (though significant), whole plant biomass was also similar for all the genotypes (Table 3). Shoot N concentration and content were also similar for the seven soybean genotypes.

The δ^15^N of soybean shoots was generally high in Amaro soil, and ranged from +3.67‰ to +4.87‰. As a result, percent N derived from fixation was low, and varied from 5.37% for Awassa-95 to 22.2% in TGx-3326-44. The amount of N-fixed by soybean genotypes in Amaro soil was very low, and ranged from 6 mg plant^−1^ for Awassa-95 to 19.5 mg plant^−1^ in TGx-3326-44. Due to the generally high N concentration in Amaro soil, the genotypes took up more N from soil than symbiosis. Soil N-uptake was high in the low-fixing Awassa-95 (101.0 mg plant^−1^), and much lower in TGx-3326-44, which fixed the most symbiotic N (Table 3).

##### Boricha

Only five soybean genotypes were evaluated in Boricha soil. The dry matter yield of shoots, roots and whole-plants were similar for all the genotypes, just as shoot N concentration and content were also not different in Boricha soil (Table 3).

As found for Amaro soil, shoot δ^15^N values were also high in Boricha soil, and ranged from +3.99‰ for Wegayen to +5.05‰ in Awassa-95. As a result, the %Ndfa values were rather low. Genotype Wegayen recorded the highest %Ndfa value of 25.5%, followed by genotypes AGS-71 (21.5%) and Coker-240 (19.7%). These 3 genotypes fixed relatively more symbiotic N than Awassa-95 and Williams (Table 3). Of the five genotypes, Coker-240 took up more N from soil (Table 3).

##### Dorebafano

Only four soybean genotypes were studied in Dorebafano soil. Plant growth, measured as shoot, root and whole-plant dry matter were similar for all four genotypes. However shoot N concentration and content differed significantly, and were much greater in AFGAT, and lower in Gishama (Table 3).

The δ^15^N of soybean shoots was very high for all four genotypes, and ranged from +4.32‰ for TGx-3326-44 to +5.77‰ in Crowford (Table 3). Due to the large δ^15^N values, the %Ndfa of plant shoots was generally very low, and ranged from 7.5% for Crowford to 26.8% in TGx-3326-44. The amounts of N-fixed by the soybean genotypes were low, and ranged from 6.9 mg plant^−1^ for Crowford to 23.1 mg plant^−1^ in TGx-3326-44 (Table 3). Relative to N-fixed, soil N-uptake by soybean genotypes was very high, with the highest uptake recorded by AFGAT and the lowest by TGx-3326-44 (Table 3).

### Plant growth and symbiotic N nutrition across soil types

Combined analysis was performed to compare plant performance across soil types. The data showed marked differences in organ and whole-plant biomass as well as symbiotic performance in soybean planted in the different soils. Amaro soil supported greater shoot growth and hence higher whole-plant biomass (Table 4). Plants in Mambuk soil showed the least organ growth, and hence lower whole-plant dry matter yield. Although shoot N concentration was greater in Boricha soil, N content was similar in all six soils.

**Table 4:** Comparison of soybean growth and symbiotic performance in soils from different locations in Ethiopia

Soil	Dry matter (DM)			Shoot N concentration	Shoot N content	δ^15^N	Ndfa	N-fixed	Soil N-uptake
	Shoot	Root	Whole-plant						
	g plant^−1^			%	mg plant^−1^	‰	%	mg plant^−1^	mg plant^−1^
Amaro	5.72 ± 0.06a	1.86 ± 0.04a	7.58 ± 0.03a	1.75 ± 0.03c	99.39 ± 2.86a	4.23 ± 0.03b	14.43 ± 0.35c	14.03 ± 0.31b	85.36 ± 2.58a
Boricha	4.34 ± 0.24bc	1.89 ± 0.12a	6.22 ± 0.35b	2.46 ± 0.09a	106.54 ± 6.93a	4.56 ± 0.07ab	18.22 ± 0.87c	19.40 ± 2.11b	87.14 ± 5.23a
Dorebafano	4.17 ± 0.06bc	1.97 ± 0.08a	6.14 ± 0.11b	2.27 ± 0.08b	95.06 ± 4.62a	5.11 ± 0.08a	16.24 ± 1.10c	15.51 ± 0.91b	79.55 ± 4.18ab
Mambuk- Forest	4.86 ± 0.57ab	1.06 ± 0.10c	5.93 ± 0.66b	2.25 ± 0.01b	107.44 ± 15.77a	1.34 ± 0.36 cd	44.38 ± 6.33b	45.97 ± 5.89a	61.48 ± 12.68b
Mambuk	3.76 ± 0.02c	0.91 ± 0.06c	4.67 ± 0.04c	2.18 ± 0.03b	80.43 ± 1.47a	1.46 ± 0.13c	57.99 ± 1.68a	46.77 ± 0.41a	33.66 ± 1.86c
Pawe	4.78 ± 0.30b	1.33 ± 0.09b	6.11 ± 0.39b	2.29 ± 0.04ab	107.15 ± 8.33a	0.82 ± 0.19d	41.84 ± 4.18b	43.95 ± 5.52a	63.20 ± 5.31b
F-statistics									
Soil locations	5.84**	29.65***	7.08**	18.52***	1.67 ns	111***	32.10***	22.10***	10.06***

Values (Mean ± SE) with dissimilar letters in a column are significantly different at *p* ≤ 0.05 (*), *p* ≤ 0.01 (**), *p* ≤ 0.001 (***)

Shoot δ^15^N values ranged from +0.82‰ for Pawe to +5.11‰ in Dorebafano. Shoot δ^15^N was highest in the soil from Dorebafano, followed by Boricha, Amaro, and Mambuk, and lowest in soil from Pawe (Table 4). As a result, higher %Ndfa values were obtained in plants grown in Mambuk, Mambuk forest and Pawe soils. The lowest shoot %Ndfa was found in Amaro soil (Table 4). The amount of N-fixed in soybean genotypes was higher in soil from Mambuk, Mambuk forest and Pawe, while the least N-fixed was in Amaro soil. Soil N-uptake varied from 33 mg plant^−1^ in Mambuk soil to 87 mg plant^−1^ in Boricha soil (Table 4).

### 2-way ANOVA comparison of growth and symbiotic performance of soybean planted in soils from two regions

A combined 2-Way ANOVA, comparison of the 11 soybean genotypes grown in soils collected from the two regions (BGR and SNNPR) was done (Table 5).

Soybean plants grown in soil from SNNPR exhibited greater shoot (4.90 g plant^−1^), root (1.78 g plant^−1^) and whole-plant (6.68 g plant^−1^) biomass when compared to soil from BGR (Table 5). There were however no significant differences in shoot N concentration and content. But shoot δ^15^N was lower in the BGR than SNNPR region. As a result, %Ndfa was much higher in soil from BGR region (Table 5). Amount of N-fixed was expectedly greater at BGR than SNNPR region, which had higher soil N uptake (Table 5).

**Table 5:** A 2-Way-ANOVA comparison of soybean genotypes for growth and symbiotic in soils collected from two regions (BGR and SNNPR) in Ethiopia. 55.

Treatments	Dry matter (DM)			Shoot N conc’n	Shoot N content	δ^15^N	Ndfa	N-fixed	Soil N-uptake
	Shoot	Root	Whole- plant						
	g plant^−1^			%	mg plant^−1^	‰	%	mg plant^−1^	mg plant^−1^
Region									
BGR	4.57 ± 0.22b	1.12 ± 0.05b	5.63 ± 0.25b	2.27 ± 0.06a	101 ± 5a	1.34 ± 0.15b	47.40 ± 2.5a	47.44 ± 2.7a	53.82.0 ± 4.6b
SNNPR	4.90 ± 0.14a	1.78 ± 0.07a	6.68 ± 0.16a	2.15 ± 0.07a	104 ± 3a	4.50 ± 0.10a	17.68 ± 1.2b	18.22 ± 1.3b	85.78 ± 3.4a
Genotypes									
AFGAT	4.66 ± 0.28 cd	1.47 ± 0.13bc	6.13 ± 0.31bc	2.62 ± 0.10a	122 ± 7a	2.89 ± 0.97 cd	34.0 ± 10.6 cd	41.1 ± 12.8a	80.4 ± 14.6ab
AGS-71	4.79 ± 0.23 cd	1.30 ± 0.11bc	6.09 ± 0.44bc	2.15 ± 0.11bcd	102 ± 4ab	2.23 ± 0.93f	43.6 ± 10.5a	44.2 ± 10.5a	57.7 ± 11.3 cd
Awassa95	6.32 ± 0.60a	1.46 ± 0.26bc	7.78 ± 0.51a	2.00 ± 0.26 cd	124 ± 20a	3.58 ± 0.62a	22.3 ± 5.0 g	28.1 ± 8.1b	95.8 ± 14.4a
Clark-63 K	4.17 ± 0.63d	1.30 ± 0.15bc	5.46 ± 0.75c	2.23 ± 0.13bc	89 ± 10b	2.94 ± 0.49 cd	31.4 ± 3.5de	26.5 ± 1.5bc	62.8 ± 9.5bcd
Coker-240	4.15 ± 0.46d	1.84 ± 0.32a	6.00 ± 0.80bc	2.62 ± 0.08a	109 ± 12ab	3.15 ± 0.59bc	28.6 ± 4.9ef	29.5 ± 3.8b	79.0 ± 12.4ab
Crowford	4.31 ± 0.14d	1.38 ± 0.12bc	5.70 ± 0.24bc	2.16 ± 0.05bcd	93 ± 2b	3.61 ± 0.63a	21.9 ± 5.1 g	20.4 ± 4.9c	72.3 ± 4.8bcd
Gishama	4.12 ± 0.13d	1.45 ± 0.25bc	5.58 ± 0.36bc	2.06 ± 0.08bcd	85 ± 3b	2.68 ± 0.88de	36.7 ± 9.5bc	31.4 ± 8.3b	53.2 ± 7.7d
Nova	5.50 ± 0.38b	1.16 ± 0.17c	6.66 ± 0.51b	1.97 ± 0.20 cd	105 ± 5ab	3.28 ± 0.42b	25.9 ± 2.5 fg	27.5 ± 3.3bc	77.5 ± 3.3abc
TGx-3326-44	5.05 ± 0.20bc	1.63 ± 0.22ab	6.69 ± 0.31b	1.85 ± 0.07d	93 ± 5b	2.19 ± 0.81f	43.8 ± 8.8a	41.3 ± 8.8a	52.1 ± 8.5d
Wegayen	4.46 ± 0.31 cd	1.59 ± 0.14ab	5.71 ± 0.39bc	2.30 ± 0.10abc	101 ± 5ab	2.43 ± 0.70ef	39.7 ± 7.0ab	41.4 ± 8.5a	60.0 ± 5.6bcd
Williams	4.53 ± 0.37 cd	1.34 ± 0.11bc	5.86 ± 0.48bc	2.37 ± 0.11ab	107 ± 8ab	3.19 ± 0.77bc	30.0 ± 7.6def	29.8 ± 7.0b	76.9 ± 13.2abc
Two-way F-statistics									
Soil location	5.65*	89.09***	24.28***	2.76 ns	0.31 ns	344.62***	132.15***	387.79***	67.79***
Genotype	8.80***	2.63**	3.64***	4.79***	2.29*	27.29***	28.13***	10.07***	4.50***
Soil location* genotype	10.05***	1.63 ns	3.90***	2.36*	1.99 ns	18.22***	19.95***	11.17***	2.84*

Values (Mean ± SE) with dissimilar letters in a column are significantly different at *p* ≤ 0.05 (*), *p* ≤ 0.01 (**), *p* ≤ 0.001 (***).

Of the 11 soybean genotypes tested in soils from the two regions, Awassa-95 produced the most shoot biomass and hence higher whole-plant dry matter. However, Gishama and Crowford recorded the lowest shoot biomass, and Clark-63 K the least whole-plant dry matter (Table 5). Genotypes AFGAT and Coker-240 showed much higher shoot N concentration. As a result, AFGAT recorded the most N content, followed by Awassa-95.

Shoot δ^15^N was higher in Awassa-95 and Crowford, and lowest in TGx-3326-44 and AGS-71. The %Ndfa values of soybean shoots ranged from 21.9 for Crowford to 43.8% in TGX-3326-44 (Table 5). The amounts of N-fixed also differed among the 11 soybean genotypes, and were much greater in genotypes AFGAT, AGS-71, TGx-3326-44 and Wegayen. The lowest amount of N-fixed was in Crowford, followed by Clark-63 K (Table 5). N-uptake from soil was higher than N obtained from symbiosis in all the genotypes tested (Table 4). But Awassa-95 took up more soil N, followed by AFGAT, with Gishama and TGx-3326-44 as the least (Table 5).

**Fig. 1:**
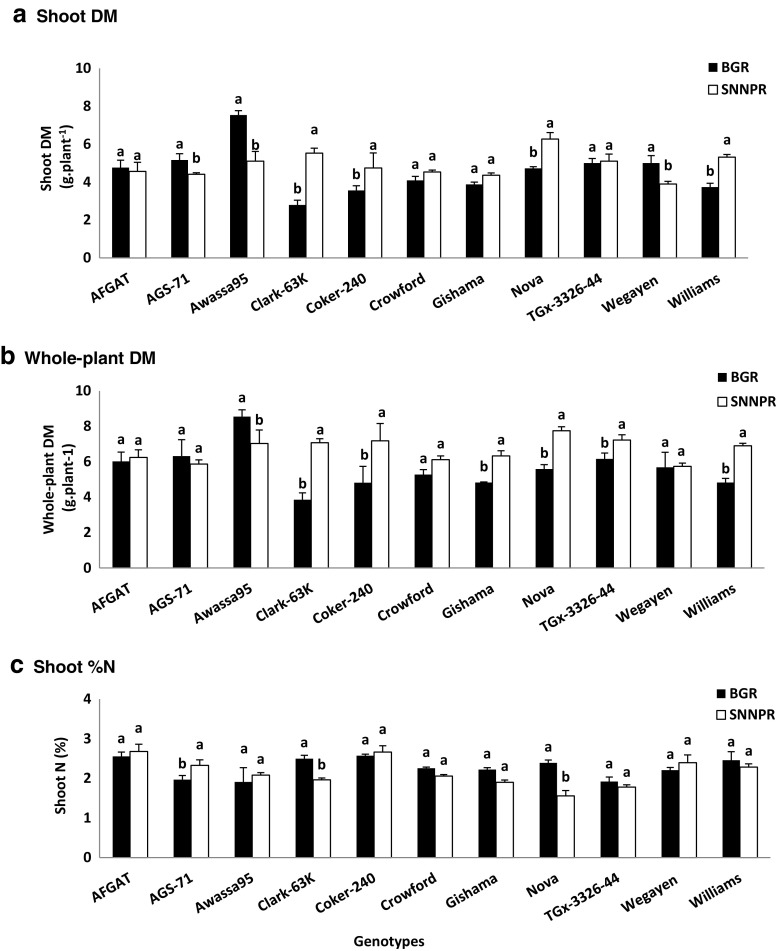

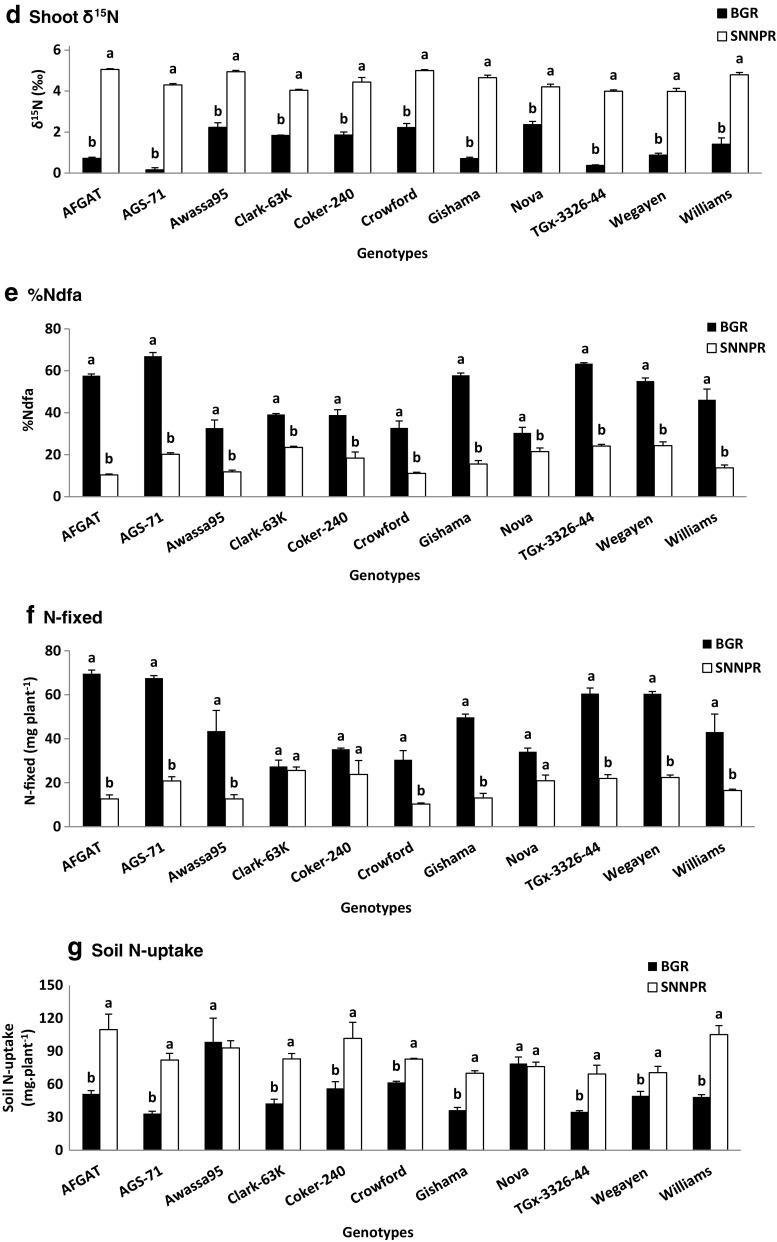
The interactive effect of soil location x genotype on A) Shoot DM B) Whole Plant DM C)Shoot %N D) δ^15^N, E) %Ndfa, and F) N-fixed and G) N-uptake of soybean plants. Bars with dissimilar letters are significantly different at *P* ≤ 0.05. Vertical line represents S.E.

The region x genotype interaction was significant for shoot and whole-plant biomass, shoot N concentration, δ^15^N, %Ndfa, N-fixed and soil N-uptake (Table 5). In general, shoot biomass of Clark-63 K, Coker-240, Nova and Williams was greater when planted in soils from SNNPR than BGR. Conversely, AGS-71, Awassa-95 and Wegayen produced more shoot biomass in soils from BGR than SNNPR (Fig. 1A). Shoot biomass of the remaining genotypes was similar in the two regions. Due to differences in shoot biomass, whole-plant dry matter was higher in six of the 11 genotypes grown in soils from SNNPR when compared to BGR (Fig. 1B). Only Awassa-95 recorded higher whole-plant biomass in soil from BGR over SNNPR. Shoot N concentrations were generally similar for the two regions, except for genotypes Clark-63 K and Nova, which showed higher shoot N concentration in soils from BGR than SNNPR (Fig. 1C).

Shoot δ^15^N of soybean genotypes was markedly greater in soils from SNNPR than BGR for all 11 genotypes (Fig. 1D). As a result, %Ndfa values were significantly lower in soybean genotypes planted in soils from SNNPR than BGR (Fig. 1E). The amount of N-fixed was also expectedly lower in all soybean genotypes raised in soils from SNNPR than BGR (Fig. 1F). The lower N_2_ fixation in soils from SNNPR region was due largely to higher soil N uptake. Nine out of the 11 genotypes took up more N from SNNPR soil when compared to BGR (Fig. 1G).

#### Correlation analysis

**Fig. 2:**
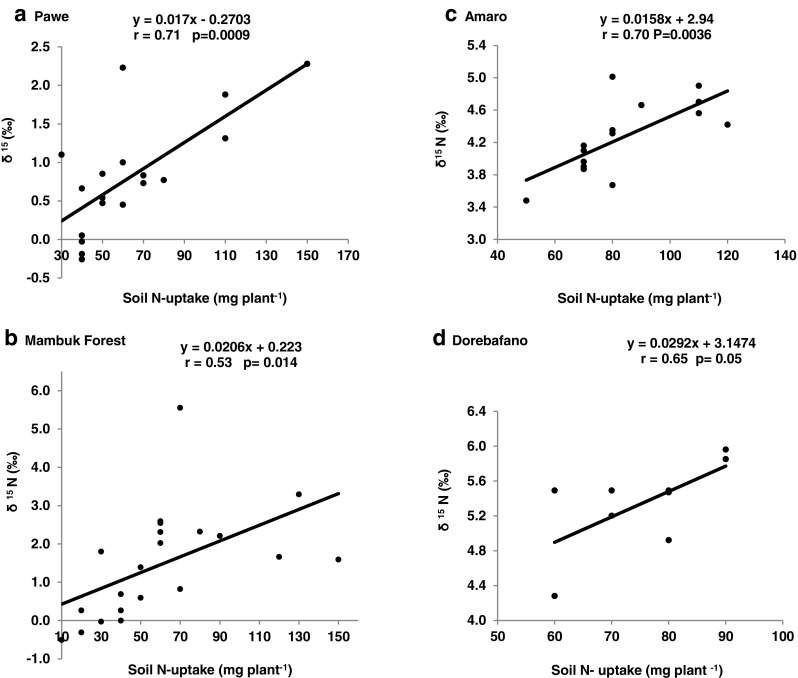
Relationship of δ^15^N and soil N-uptake of soybean genotypes grown under glasshouse condition in soils from different locations in Ethiopia

**Fig. 3:**
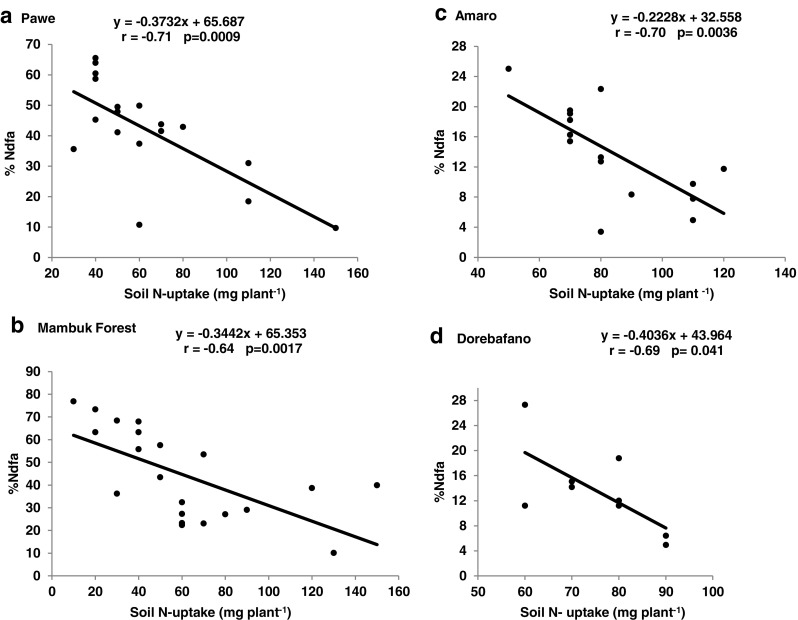
Relationship of %Ndfa and soil N-uptake of soybean genotypes grown under glasshouse condition in soils from different locations in Ethiopia

Whether dealing with soil from Pawe, Mambuk forest, Amaro, or Dorebafano, shoot δ^15^N was significantly correlated with soil N uptake (Fig. 2 A, B, C and D). As a result, percent N derived from fixation was markedly correlated with soil N uptake by soybean grown in Pawe, Mambuk forest, Amaro and Dorebafano soils (Fig. 3 A, B, C and D).

## Discussion

This study assessed the performance of soybean symbiosis with native rhizobial populations in soils from different locations in Ethiopia, using the ^15^N natural abundance technique. The accuracy of the ^15^N natural abundance technique depends on the levels and uniformity of the ^15^N isotope in the soil and the choice of B value. δ^15^N values of reference plants > +6.0‰ are preferred, even though values as low as +2‰ can still be useful, as the δ^15^N is less affected by temporal and depth variations in agricultural soils (Herridge and Rose 2000; Okito et al. 2004; Unkovich et al. 2008). It has been recommended that more than one non-N_2_-fixing reference plants be used for estimation of N_2_ fixation using the ^15^N natural abundance method (Unkovich et al. 2008). Therefore, 3 non-legume reference plants [*Sorghum bicolour* (L.), *Eragrostis tef* and *Triticum aestivum*] were used in this study, with average δ^15^N values that varied from +2.72 to +6.33‰, a range suitable for estimating soil N-uptake by legumes (Unkovich et al. 2008).

The 1-Way ANOVA found significant differences among the soybean genotypes planted in different soils (Table 3). While this variation could be attributed to genetic differences among the genotypes, soil N uptake and/or rhizobial strain effect is equally important (Herridge and Rose 2000). Other studies have similarly found variations in plant growth, nodulation and N_2_ fixation of soybean genotypes due to differences in N_2_-fixing efficiency of the microsymbiont (Pule-Meulenberg et al. 2011; Salvucci et al. 2012).

There was a location-specific effect of soil on plant growth and symbiotic N nutrition in soybean. For example, whole-plant biomass was greater in soybean planted in soil collected from Amaro, followed by Boricha, Dorebafano, Pawe, and Mambuk (Table 4). The soil properties of these locations were variable due largely to differences in nutrient concentrations (Table 1). Soybean plants grown in soil from Boricha had the highest shoot N concentration, with the lowest in soil from Amaro. Shoot N content was comparable across soil types although plants grown in soils from Pawe, Boricha and Mambuk forest had relatively higher shoot N content when compared to those from the other soils (Table 4). Shoot δ15N values ranged from +0.82‰ for Pawe to +5.11‰ in Dorebafano. Consequently, greater %Ndfa of soybean was obtained in plants grown in Mambuk soil, followed by Pawe. The lowest %Ndfa value was in Amaro soil (Table 4). The amount of N-fixed followed a similar trend. Though soils from Mambuk had medium nitrate levels they supported high levels of N_2_ fixation in soybean (Table 4). It has been shown that some soybean genotypes are capable of developing nitrate- tolerant symbioses especially when the rhizobial population is high (Herridge and Rose 2000).

Soybean plants grown in soils from the BGR region recorded the lowest δ^15^N values, and as a result derived the highest N from fixation when compared to SNNPR (Table 5). This could be attributed to the presence of high population of compatible very effective indigenous soil rhizobia that formed functional N_2_-fixing root nodules. Though plant biomass was greater for soybean grown in SNNPR soils, they recorded low %Ndfa. This could be due to the fact that the plants were meeting their N requirements from soil since that location (Amaro) showed moderate levels of soil nitrate and high organic C concentration (Amaro) (Table 1). In high nitrate soils, most legumes including soybean, tend to take up moderate to large amounts of soil N at the expense of fixation, often resulting in low %Ndfa and reduced amounts of N-fixed (Herridge and Rose 2000). A recent study also found nitrate inhibition of nodulation and N_2_ fixation in nodulated soybean receiving 5 mM NO_3_^−^ (Mbah and Dakora 2017). It has been found that large amounts symbiotic N is produced if the legume demand for N exceeds soil N supply, under optimal conditions (Salvagiotti et al. 2008).

The significant interaction between soil and soybean genotype for plant DM, shoot N concentration, δ^15^N, %Ndfa, N-fixed and soil N-uptake clearly indicate the modulating effect of soil factors on legume N_2_ fixation (Table 5). Soybean genotypes Coker-240, Clark-63 K, Williams, Nova, TGx-3326-44 and Gishama had greater whole-plant biomass values in SNNPR than BGR soils (Fig. 1 B). All genotypes studied also showed significantly greater δ^15^N values in SNNPR compared to BGR soils (Fig. 1 D), which led to higher N derived from fixation in soils from the BGR. For example, 8 of the 11 soybean genotypes recorded higher %Ndfa values in BGR compared to SNNPR soils, a clear indication of more effective rhizobia in the former location. Soybean is estimated to have %Ndfa ranging from 0 to 97% and N contribution of 0 to 450 kg per hectare, depending on the host-rhizobia symbiosis, the strength of the symbiotic N sink, the concentration of endogenous soil N, and other soil factors (van Kessel and Hartley 2000; Unkovich and Pate 2000). Thus, the wide variation in %Ndfa of soybean in this study could be linked to these factors. Not only was soil N uptake greater in SNNPR region, but N uptake by individual soybean genotypes was also large, ranging from 53 mg plant^−1^ in Nova to 96 mg plant^−1^ in Awassa-95. In fact, the negative correlation obtained between soil N uptake and %Ndfa (Fig. 3 D) clearly indicate that soil N was a major factor affecting soybean N_2_ fixation in the soils used. It has also been reported that N_2_ fixation is often underestimated by about 24% when below-ground N is ignored in nodulated roots (Unkovich and Pate 2000; Salvagiotti et al. 2008). As this study did not include N in nodulated roots, the amount of N-fixed in the soybean genotypes could have been underestimated.

Although this study did not assess the existing cropping systems at the sites of soil collection, both legume-based and cereal-based rotations have been reported to alter the populations of soybean rhizobia in soil, with the former stimulating more rhizobial growth than the latter (Kumar et al. 2017). Thus, some of the differences in nodulation and N_2_ fixation could possibly be attributed to the type of rotations used by farmers in the BGR and SNNPR regions of Ethiopia, especially where there were location-specific effects of soil on symbiotic performance. Interestingly, even where there was no history of soybean cultivation as found at Mambuk forest soil, there were still native rhizobia that could nodulate and fix N_2_ in soybean, probably suggesting alternate host plants that haboured rhizobia in their rhizospheres in the absence of soybean, the homologous host. In a separate study, rhizobia isolated from root nodules of soybean planted in these soils were found to differ in their symbiotic performance (data not shown), and this could also help to explain the variations found in soybean nodulation and N_2_ fixation in this study.

Although soil factors such as P, CEC, and EC revealed no correlation with soybean symbiotic performance, shoot δ^15^N was significantly correlated with soil N uptake by soybean in soil from Pawe, Mambuk forest, Amaro, or Dorebafano (Fig. 2 A, B, C and D), suggesting greater dependence on soil N than symbiosis for N nutrition in those soils. As a result, percent N derived from fixation was reduced by uptake of soil N from all four locations through inhibition of N_2_ fixation (Fig. 3 A, B, C and D; see Ayisi et al. 2000).

Taken together, the differences in symbiotic functioning among soybean genotypes could be attributed to a range of factors, which include i) the presence and/or competitiveness of native soybean rhizobia in the soil, ii) the level of strain symbiotic efficacy, iii) mineral concentrations in soil, especially N (Dakora and Keya 1997), and iv) the presence of bacteriophages in soil (Msimbira et al. 2016). There was also clear evidence of mineral N inhibition of N_2_ fixation in soybean planted in soils from various sites.
